# Patients With Culturally Diverse Backgrounds and Their Experiences With an Interpretation Intervention Combining Video Interpretation, Extended Consultation Time, and Interdisciplinary Collaboration in a Medical Spine Clinic: A Qualitative Study

**DOI:** 10.1002/msc.70199

**Published:** 2026-03-02

**Authors:** Ann‐Louise Larsen, Janni Tran, Knud Ryom, Stine Aalkjær Clausen, Anne Mette Schmidt

**Affiliations:** ^1^ Digitalisation and IT Central Denmark Region Aarhus Denmark; ^2^ Center for Nursing and Rehabilitation Research VIA University College, Campus Aarhus N Aarhus Denmark; ^3^ Department of Public Health, Applied Public Health Research Aarhus University Aarhus Denmark; ^4^ Medical Diagnostic Centre University Clinic for Innovative Patient Pathways, Regional Hospital Central Jutland Silkeborg Denmark; ^5^ The Danish Multiple Sclerosis Hospitals Ry and Haslev Denmark

**Keywords:** back pain, communication barriers, emigrants and immigrants, ethnicity, telemedicine, translations

## Abstract

**Introduction:**

Back pain is caused and driven by a complex interplay of multiple factors, including cultural background. In healthcare, patients from culturally diverse backgrounds may face barriers such as language difficulties and a lack of cultural sensitivity, which can exacerbate health disparities.

**Objective:**

How do patients with culturally diverse backgrounds and back pain experience an interpretation intervention combining video interpretation, extended consultation time, and interdisciplinary collaboration?

**Methods:**

This qualitative study was conducted at a Danish medical spine clinic. Participant observation and previous research informed the semi‐structured interview guide. Individual interviews were conducted with seven patients from culturally diverse backgrounds (four women, aged 30–62 years, representing three different countries) with interpreter support. The interviews were audio‐recorded, transcribed verbatim, and analysed using thematic network analysis.

**Results:**

Patients reported that video interpretation facilitated communication and respected cultural values and assumptions, with some women highlighting the importance of interpreter gender choice. The extended consultation time provided a clear structure, reinforced communication, enhanced disease‐related knowledge, and supported patients' expectations. Finally, interdisciplinary collaboration further strengthened communication and disease‐related knowledge, aligned care with patients' expectations, and contributed to a coherent structure, while emphasising the need to balance structured consultations with opportunities for patient expression.

**Conclusion:**

This study provides novel insights into how organisational adaptations support culturally responsive, person‐centred management. Integrating video interpretation, extended consultation, and interdisciplinary collaboration promotes equitable care, enhances patient engagement, and highlights the value of organisational health literacy. Future improvements include involving patients in choice of interpreter and ensuring space for patient expression.

## 1 Introduction

Globally, low back pain affected 619 million people in 2020, making it the leading cause of years lived with disability (GBD 2021 Low Back Pain Collaborators 2023) and a major public health challenge worldwide (Foster et al. 2018; Hartvigsen et al. 2018). The development and persistence of back pain, along with the associated disability, result from an intricate interplay of biophysical, genetic, psychological, social, and lifestyle factors, as well as comorbidities (Hartvigsen et al. 2018; Hoy et al. 2010; Vlaeyen et al. 2018). Consequently, back pain necessitates a holistic approach and represents one of the most challenging conditions for patients, health professionals, and stakeholders to manage (Haldeman et al. 2012; Vlaeyen et al. 2018).

Cultural background and ethnicity play a critical role in shaping patients' experiences of back pain (Hoy et al. 2010; Singh et al. 2018). Language barriers and limited cultural sensitivity in clinical interactions may cause patients to feel misunderstood or disrespected, which can contribute to strained patient‐health professional relationships, reduced treatment satisfaction, heightened pain and anxiety, and ultimately poorer health outcomes (Brown et al. 2021; Slade and Sergent 2024; Sodemann et al. 2015). These dynamics highlight the importance of culturally responsive management strategies to mitigate health disparities and improve health outcomes among patients from culturally diverse backgrounds.

Given ongoing and increasing global migration (Danmarks 2026), patients with limited proficiency in the official language present a rising challenge to healthcare systems in their countries of residence (Davidsen et al. 2022). This highlights the clear need to develop healthcare interventions that are culturally and linguistically inclusive (Kletecka‐Pulker et al. 2021).

In response, an intervention termed the interpretation intervention, comprising video interpretation, extended consultation time, and interdisciplinary collaboration, has previously been developed in a Danish medical spine clinic (Schmidt et al. 2024). While health professionals have reported positive evaluations of this intervention (Schmidt et al. 2024), patients' experiences remain unexplored.

Existing research on video interpretation interventions is limited with most studies focussing on management and/or health professionals' perspectives (Eltayeb et al. 2025; Feiring and Westdahl 2020; Mottelson et al. 2018; Schmidt et al. 2024). Although several reviews have examined patients' preferences and satisfaction with different interpretation modes (face‐to‐face, telephone, or video) (Haralambous et al. 2019; Heath et al. 2023; Joseph et al. 2018), no study has, to our knowledge, qualitatively explored patients' experiences with video interpretation interventions.

In summary, little is known about how patients with culturally diverse backgrounds experience interventions designed to reduce health disparities and promote culturally responsive management. Understanding these experiences is critical for evaluating such interventions and improving culturally responsive management. Accordingly, this study explores how patients with culturally diverse backgrounds and back pain experience an interpretation intervention combining video interpretation, extended consultation time, and interdisciplinary collaboration.

## 2 Methods

### 2.1 Design

This deductive qualitative study focused on three predefined components of the interpretation intervention. Both interviews and analysis were guided by these components, rather than allowing unanticipated themes to drive data collection or coding. This study was inspired by the Standards for Reporting Qualitative Research (O'Brien et al. 2014).

### 2.2 Setting

This study was conducted at a medical spine clinic at Silkeborg Regional Hospital, Central Denmark Region, Denmark. The clinic serves as a specialised diagnostic unit for patients with back pain. Adult patients in the region who do not improve with management in the primary sector and who have no indication for surgery may be referred to the medical spine clinic. Annually, around 5000 patients are referred by general practitioners or specialists; of these, approximately 60 patients (≈1%) have culturally diverse backgrounds.

### 2.3 Intervention

The interpretation intervention is a tailored intervention for patients with culturally diverse backgrounds and back pain, comprising three components: (1) video interpretation facilitated via video conferencing equipment (computer, camera, and microphone; Cisco) connected to an interpreter from an external agency through Rooms, a secure online platform; the interpreter was not necessarily professionally certified, (2) extended consultation time with a duration of up to 2 hours, and (3) interdisciplinary collaboration jointly delivered by a rheumatologist and a physiotherapist. The intervention has been described in detail previously (Schmidt et al. 2024).

### 2.4 Participants

Patients were recruited consecutively in two phases during a predefined 10‐week period (March and May 2024). Therefore, the final sample comprised all eligible patients who provided consent during this period.

In the first phase, clinicians (rheumatologists or physiotherapists) informed patients who were deemed capable of participating in interviews at the end of their consultation. Patients who expressed interest were provided with written information materials, outlining the purpose of the interview, the conditions for participation, and a consent form in 12 languages (Afghan (Dari), Afghan (Pashto), Arabic, Bosnian, English, Latvian, Lithuanian, Polish, Romanian, Tamil, Turkish, and Ukrainian). Communication at this phase was supported by the same interpreter, who was present via video during the consultation.

In the second phase, two authors (AL and JT) managed further communication, arranging the time and place of the interviews via SMS or email using DeepL.com, an AI‐powered language platform.

### 2.5 Data Collection

Two authors (AL and JT) developed a semi‐structured interview guide based on prior participant observations of four consultations involving the interpretation intervention and two standard consultations, and previous research in the setting (Schmidt et al. 2024) before discussion in the wider author group. The semi‐structured interview guide focused on the three components of interpretation intervention, enabling in‐depth exploration of patients' perspectives.

To prevent patient fatigue immediately following the two‐hour consultation in the medical spine clinic, interviews were conducted 5–12 days later, allowing time for rest and reflection on the consultation prior to participation in the interview. The interview setup was adapted to patient preference: in‐person interviews (*n* = 1) and telephone interviews (*n* = 6).

Interpreters for the interviews were recruited through the same external agency providing interpretation services during the consultation; verbal consent for audio recording was obtained. Interviews were conducted jointly by two authors (AL and JT), lasted 15–43 min, and were audio‐recorded and transcribed verbatim. Transcripts were checked against recordings to verify accuracy and anonymised using pseudonyms. All data were stored in a secure, password‐protected system accessible only to the research team.

### 2.6 Data Analysis

A thematic network analysis, based on Attride‐Stirling's *Thematic Networks: An Analytic Tool for Qualitative Research*, was employed (Stirling 2001). Two authors (AL and JT) independently identified and organised themes hierarchically, beginning with the global theme at the highest level, which captured the overarching patterns in the data, followed by organising themes at the mid‐level, and concluding with basic themes at the lowest level. The two authors then compared the results and resolved discrepancies through discussion with the wider author group. Analytic notes documenting coding decisions, theme development, and reflections on researcher influence were maintained throughout to support transparency, confirmability, and an audit trail.

### 2.7 Researcher Characteristics, Reflexivity and Techniques to Enhance Trustworthiness

Two early‐career researchers (AL and JT) with Public Health backgrounds and no prior familiarity with the population conducted interviews and led data analysis, providing an outsider perspective. The wider author team included a senior qualitative researcher (KR) experienced in health sciences research, including ethnic minority health, a physiotherapist (SAC) with clinical expertise in the population, pathway, and interpretation intervention who assisted with recruitment, and a researcher‐physiotherapist (AMS) experienced in innovative patient pathways.

AL and JT reflected on how their backgrounds could shape interviews and interpretations, documented these reflections in analytic notes, and discussed interpretations with the wider author group, allowing critical examination of potential biases and considerations of alternative explanations.

Participant observations of consultations prior to developing the semi‐structured interview guide enhanced contextual understanding while maintaining analytical independence.

Trustworthiness was further supported through independent coding by two researchers, comparison of themes, probing participants' responses, revisiting interpretations, and regular discussions within the wider author group. To ensure that interpretations accurately reflected participants' intended meanings, analytic decisions were continuously reviewed and challenged by author group members with both clinical expertise and qualitative research experience, and all key decisions were documented in an audit trail. Credibility was reinforced by triangulation of observations and interviews, and the use of a semi‐structured interview guide informed by observations and prior research in the setting. These procedures systematically verified that findings reliably represented participants' experiences. Transferability was facilitated by detailed descriptions of the study setting, recruitment, intervention, and analytic procedures.

## 3 Ethical Approval

Informed consent was obtained from all patients and interpreters included in the study. According to Danish regulations, the study did not fall within the scope of the Danish Medical Research Involving Human Subject Act (§14) (Retsinformation 2020). The study adhered to the ethical principles outlined in the Declaration of Helsinki.

## 4 Results

Data collection and analysis were conducted from March to June 2024. In total, nine patients were invited to participate during the 10‐week inclusion period, of whom seven consented. The sample included four women and three men, aged 30–62 years, from three different countries, with residency in Denmark ranging from 2 to 12 years (Table 1).

**TABLE 1:** Characteristics of the seven patients included in the study.

Patient	Age	Gender	Country of origin	Years lived in Denmark
1	50	Male	Syria	8
2	31	Female	Ukraine	2
3	30	Female	Syria	8
4	62	Female	Ukraine	2
5	32	Female	Kurdistan	6
6	50	Male	Syria	12
7	38	Male	Syria	9

In line with thematic network analysis, the global theme identified was Experiences of the interpretation intervention, and the three organising themes deducted were Video interpretation, Extended consultation time, and Interdisciplinary collaboration. Each organising theme included some of the five basic themes as follows:

- Video interpretation: communication, and cultural values and assumptions.
- Extended consultation time: communication, disease‐related knowledge, expectations, and structure.
- Interdisciplinary collaboration: communication, disease‐related knowledge, expectations, and structure.

These basic themes are explicitly mentioned in the narrative below and illustrated with interview quotations.

### 4.1 Video Interpretation

This organising theme included two basic themes, namely communication, and cultural values and assumptions.

Several patients reported that video interpretation was a valuable tool that facilitated effective communication. One patient stated:

I think it was very good to use video interpretation for the consultation

(P5)

This response indicated that the patient's expectations were met, reflecting a high level of satisfaction and appreciation for video interpretation.

Patients emphasised that the presence of an interpreter facilitated understanding of health professionals' communication and ensured that their own perspectives were accurately conveyed. As one patient explained:

Yes, because of the interpreter, I understood everything the health professionals said, and I think it worked the other way too

(P1)

Patients highlighted several practical benefits of video interpretation, including enhanced presence, flexibility, and clarity in a situation with linguistic and cultural barriers. One patient described:

It was like the interpreter was sitting right next to me during the consultation. So, I could explain and demonstrate what I was referring to, and both the interpreter and health professionals could understand everything

(P2)

This highlights how video interpretation fosters a sense of presence and direct engagement, enhances communication, and supports mutual understanding between patients and health professionals.

The option to deactivate the camera during physical assessments was intentionally incorporated to enhance cultural values and assumptions related to privacy and was particularly appreciated by patients. One patient explained:

Video interpretation is better because (…) you can feel a bit embarrassed, and it can be uncomfortable when you have to undress for the consultation, so it's nice that you can turn off the camera and they can still interpret

(P3)

Some of the female patients also indicated a preference regarding interpreter gender, reflecting cultural values and assumptions. They stated:

I would prefer a female interpreter

(P3)

If there are personal matters, like sexually transmitted diseases or something between a boy and a girl … In our tradition in Arab regions, it’s a bit embarrassing to share these things with others. Maybe it doesn’t matter as much in Denmark, but for us, it is embarrassing

(P1)

These statements reflect cultural values and assumptions that emphasise privacy and discretion, particularly regarding sensitive topics. Some female patients reported feeling greater comfort and trust when the interpreter's gender matched their preference, whereas mismatches could lead to withholding information, potentially affecting subsequent management. Cultural differences regarding visual communication and privacy may explain both the appreciation of camera control and interpreter gender preferences.

Overall, video interpretation emerged as a valuable tool that facilitated clear communication, accommodated cultural values and assumptions, and fostered trust, comfort, and engagement, thereby supporting patient‐centred management. An exception to the positive experiences was the need from some female patients to have an influence over the choice of the interpreter's gender.

### 4.2 Extended Consultation Time

This organising theme encompassed four basic themes, including communication, disease‐related knowledge, expectations, and structure.

Patients perceived the extended consultation time (structure) as valuable and necessary, allowing for a thorough assessment and sufficient time to address all relevant issues related to their health condition. This helped ensure that the consultation met the patients' expectations. One patient stated:

I think two hours is enough time. They had plenty of time to examine me and my issues, so it wasn't just questions

(P3)

Some patients acknowledged that the two‐hour consultation was necessary to complete the required assessments. One patient highlighted:

In relation to the assessments that the health professionals had to do, the duration was appropriate. They had to examine my whole body, so I don't think it was too long for what I needed

(P7)

Another patient supported this but felt that the consultation was lengthy (structure) but still met the individual's expectations:

I'm glad I was referred to the medical spine clinic. Even though the consultation took a lot of time, I'm still really happy because the health professionals got all the information about my problem

(P1)

Several patients reported that the extended duration contributed to a greater sense of safety, provided a clearer understanding of their pain and health condition (disease‐related knowledge), and enhanced the structure of the consultation by allowing sufficient time for all assessments, supporting their expectations. One patient expressed:

I have gotten a good explanation of my pain and how to manage it

(P7)

These findings suggest that the additional consultation time facilitated not only effective communication but also a comprehensive assessment of the patient's health condition, underscoring the importance of allocating sufficient time to support the patients' disease‐related knowledge, promote a diagnosis and prognosis, and inform an appropriate management strategy. Importantly, patients emphasised the value of understanding their condition and future management, enabling them to feel more confident and involved.

Overall, extended consultation time allowed for thorough assessment, meaningful communication, and comprehension of the patient's disease‐related knowledge and management strategies, and a clear consultation structure fostering confidence, trust, and a sense of security, supporting expectations.

### 4.3 Interdisciplinary Collaboration

This organising theme included several basic themes, namely communication, disease‐related knowledge, expectations, and structure.

As a part of the interpretation intervention, both the rheumatologist and the physiotherapist were present during the consultation, facilitating interdisciplinary collaboration and dynamic interaction. Patients reported positive experiences, highlighting professionalism, attentiveness, and a holistic approach in which patients felt acknowledged and understood. One patient described:

It worked really well, and I think it's a good idea because one asked questions, the other assessed me, and finally they explained to me what was wrong and what I should do going forward. I've been to my own doctor many times, where I didn't understand my problem and what was wrong with me, but at the medical spine clinic, I understood what was wrong with me

(P3)

The interdisciplinary collaboration enhanced communication and supported patients' disease‐related knowledge, including an understanding of the proposed management strategies. In contrast to patients' experiences with their general practitioner, this approach facilitated a clear consultation structure, fostering a sense of respect and support from health professionals. Another patient described:

I’ve had the feeling that the health professionals were good. They respected me and wanted to help me with my problems

(P5)

Several patients reported that the interdisciplinary collaboration (structure) fostered a calm and empathetic consultation approach, allowing assessments to be conducted at a comfortable pace, even when procedures were painful or challenging. As one patient explained, this supported their expectations:

I felt comfortable during the consultation. The health professionals were really nice, and during the assessments, they were quiet and calm, which was good because they knew it could be painful to do these assessments. So, they took it all nice and easy, and I had a really good experience

(P3)

Some patients noted that the structured nature of the consultation (structure) could feel rigid and limit opportunities to incorporate their own perspectives. As one patient remarked:

It was more one‐way communication, where they told me and asked about the things they wanted to know […] they had their own plan

(P4)

This highlights a potential area for improvement. Although the structured approach is generally valued for providing clarity and thoroughness, it is important to balance it with a more flexible approach that allows patients to express their views and concerns more freely.

Interdisciplinary collaboration also increased the likelihood that patients perceived their expectations for future management as being met, and in some cases exceeded. Discussing future management was essential, as patients consistently emphasised the importance of clarity and continuity in their ongoing and future management. One patient expressed satisfaction with having their preferences acknowledged and addressed:

The staff referred me to rehabilitation, which I really wanted, and it was free. Initially, my work paid for my treatments, and later I had to pay for some massage for my pain. But this time, I've been referred to a free rehabilitation programme, which I think is great

(P2)

Overall, the interdisciplinary collaboration facilitated effective communication, enhanced patients' disease‐related knowledge, supported their expectations, and was largely supported by the structure of the consultation. Together, these elements support understanding, trust, and a sense of respect. However, the structure was also perceived as a hindrance to the patient's ability to actively share their perspective, highlighting the importance of balancing structure with flexibility.

## 5 Discussion

This study explored the experiences of patients with culturally diverse backgrounds and back pain who participated in an interpretation intervention combining video interpretation, extended consultation time, and interdisciplinary collaboration at a Danish medical spine clinic. Patients reported that video interpretation facilitated clear communication while respecting cultural values and assumptions. Extended consultation time allowed thorough assessments and meaningful communication, fostering confidence and a sense of security. Additionally, interdisciplinary collaboration enhanced communication, supported disease‐related knowledge, aligned with patient expectations, and was largely supported by a structured consultation approach. Collectively, these factors contributed to patients feeling better informed, more engaged, and more trusting of health professionals, indicating improved understanding of their health condition and potential management strategies. Together, these findings suggest that such organisational adaptations can foster more comprehensive, patient‐centred management and support equitable management for patients with limited proficiency in the official language. Potential improvements include involving patients in choosing the interpreter's gender and ensuring that the consultation structure allows for their full expression.

This study foregrounds the experiences of patients with culturally diverse backgrounds, an underrepresented perspective in the existing literature, thereby contributing novel insights into culturally responsive interventions. While prior research has mainly been centred on management and health professionals' perspectives (Eltayeb et al. 2025; Feiring and Westdahl 2020; Hill et al. 2023; Madden et al. 2020; Mottelson et al. 2018; Schmidt et al. 2024), the present findings complement these accounts.

Previous reviews report mixed findings regarding interpreter modality; some indicate that in‐person interpreters yield higher patient satisfaction (Haralambous et al. 2019; Heath et al. 2023), while others have found satisfaction among patients to be comparable between video and in‐person interpretation (Joseph et al. 2018). In the present study, patients were given the opportunity to provide their explanatory perspectives on the value of video interpretation. They highlighted video interpretations in maintaining discretion and respecting cultural values and assumptions, particularly through technical features such as the option to disable visual communication. This technical feature has also been identified as positive in prior research examining both management and health professionals' perceptions of video interpretation (Feiring and Westdahl 2020). Our prior evaluation of health professionals' perspectives on video interpretation reported practical advantages in communication, notably its facilitation of eye contact and body language observation, which enhanced communication among patients, interpreters, and health professionals (Schmidt et al. 2024). Another study on health professionals' attitudes towards video interpretation reported overall satisfaction but noted concerns about training and challenges in critical situations (Mottelson et al. 2018). These concerns were not evident in a previous study in an emergency department (Eltayeb et al. 2025) nor among our health professionals (Schmidt et al. 2024), both indicating a preference for video interpretation compared to the phone‐based interpretation.

Collectively, these perspectives suggest that integrating video interpretation within supportive organisational adaptations may enhance informational clarity, trust, and patients' sense of being understood, thereby strengthening patient‐centred management.

In the present study, extended consultation time was consistently described as a key factor in patient‐centred management, enabling thorough assessment, meaningful communication, and improved understanding of their health condition and management strategies. It also fostered patients' feelings of being heard and respected, emphasising the importance of relational aspects. These findings are built upon evidence from a study of refugees using video interpreters in primary care walk‐in clinics, which demonstrated that longer consultations were associated with higher levels of patient‐centred communication, as assessed by two independent raters (Hill et al. 2023). One additional study on primary care delivery for patients with complex medical, behavioural, and social needs also enhanced the need for long consultations to support communication (Madden et al. 2020). Our previous evaluation of health professionals' perspectives on the interpretation intervention similarly reported that the extended consultation time allowed sufficient time to consider all biophysical, psychological, and social factors important to the patient (Schmidt et al. 2024). Taken together, these findings indicate that adequate consultation time is not merely a structural adjustment but a key factor for providing equitable and culturally responsive patient‐centred management.

Interdisciplinary collaboration was perceived to foster understanding, trust, and respect, thereby enhancing cultural responsiveness among the patients in the present study, who valued the holistic assessment provided by the interdisciplinary team. Although interdisciplinary collaboration is recognised for supporting holistic patient management, no studies have explored patients' perspectives on interdisciplinary collaboration among culturally diverse groups, despite ethnicity intensifying challenges faced by patients with complex health conditions (Cabanilla et al. 2025; Grasso et al. 2025; Hoy et al. 2010; Kucukkaya et al. 2025; Singh et al. 2018; Velu et al. 2024). An integrative review of patients with chronic conditions but without focus on ethnicity reported positive patient experiences with interprofessional collaboration, supporting that it is appropriate for chronic disease management (Davidson et al. 2022). From the healthcare professionals' perspective, interdisciplinary collaboration is essential for equitable management in ethnically diverse populations with complex health conditions (Cabanilla et al. 2025; Grasso et al. 2025; Kucukkaya et al. 2025; Madden et al. 2020; Velu et al. 2024). This aligns with findings from our previous evaluation of the same intervention that found that interdisciplinary collaboration was valued by health professionals as it enabled a biopsychosocial approach, facilitated complementary roles during the consultation, and reduced patient fatigue by avoiding repeated information (Schmidt et al. 2024). By foregrounding the patient perspective, the present study extends the findings that interdisciplinary collaboration is not only experienced as valuable for health professionals but also strengthens patients' experience of respect, trust, and coherence, highlighting its value for patient‐centred, culturally responsive management.

Our findings align with the concept of organisational health literacy, defined as how healthcare services, organisations, and systems make health information and resources available and accessible to people with varying health literacy levels (Trezona et al. 2017). Ethnic or linguistic minority groups often face challenges related to limited health literacy, which likely contributes to the causal pathway between social determinants and individual health outcomes as well as social health inequalities at the societal level (Aaby et al. 2022). Thus, interventions that improve health literacy or mitigate the impact of limited health literacy may help reduce social health disparities. Organisations that are health‐literate ensure that most of the population can access and benefit from services and provide additional support to minorities (Aaby et al. 2022). The patients' experiences illustrate how the interpretation intervention promoted organisational health literacy in daily clinical practice. Video interpretation supported communication and respected cultural values and assumptions. Extended consultation time provided a clear structure, reinforced communication, enhanced disease‐related knowledge, and supported patients' expectations. Interdisciplinary collaboration further strengthened communication and disease‐related knowledge, aligned care with patients' expectations, and contributed to a coherent structure. Together, these elements demonstrate how targeted organisational adaptations can make care more accessible, patient‐centred, and culturally responsive.

This study possesses several strengths. First, the interview guide was informed by prior participant observations of consultations, which strengthened the interview guide by ensuring that questions were grounded in clinical practice and addressed the most relevant components of the interpretation intervention. Second, semi‐structured interviews allowed for in‐depth exploration of patients' experiences while maintaining flexibility to pursue emergent themes, resulting in a nuanced understanding of their perspectives. Third, recruitment and communication were carefully adapted to patients' linguistic and cultural needs, with translated recruitment materials and interpreter support throughout the interviews, thereby enhancing inclusivity and data quality. Fourth, the study included a heterogeneous sample in terms of gender, country of origin, and years lived in Denmark, reflecting clinical diversity and increasing the transferability of findings. Finally, the thematic network analysis provided a structured and systematic approach to data analysis, enabling transparent interpretation.

This study also has some limitations. It was conducted in a single clinical setting with a small sample size, which may limit the generalisability of findings to other contexts and populations. Although the sample was intentionally heterogeneous, the perspectives of patients from other cultural or linguistic backgrounds may not be fully captured. Recruitment through clinician referrals may have introduced selection bias by favouring more engaged patients. Moreover, interpreters were recruited from an external agency and were not necessarily professionally certified, which may have introduced variation in interpreting quality, potentially affecting both the consultation experience and the richness of the interview data. Finally, data sufficiency was pragmatically constrained by the predefined inclusion period. Nevertheless, the interviews conducted provided rich and meaningful insights, enabling us to explore the three components of the interpretation intervention.

## 6 Conclusion

This study offers novel insights into how patients with culturally diverse backgrounds and back pain experience an interpretation intervention comprising video interpretation, extended consultation time, and interdisciplinary collaboration. From the patients' perspective, these organisational adaptations enhance communication, foster trust, and improve understanding of their health condition and future management strategies. Future improvements include involving patients in choosing the interpreter's gender and increasing awareness of consultation structures that allow space for patient expression. By centring patient perspectives, the study underscores the critical role of culturally responsive, person‐centred management and emphasises the value of integrating organisational health literacy principles to support equitable and culturally responsive management in clinical practice.

## Author Contributions

All authors contributed to the study conception and design. Material preparation, data collection and analysis were performed by Ann‐Louise Larsen and Janni Tran and discussed in the wider author group. The first draft of the manuscript was written by Ann‐Louise Larsen and Janni Tran and the wider author group commented on previous versions of the manuscript. All authors have read and approved the final manuscript.

## Funding

Medical Diagnostic Centre, Regional Hospital Central Jutland, financed the salaries of the interpreters used for the interviews.

## Ethics Statement

Informed consent was obtained from all patients and interpreters included in the study. According to Danish regulations, the study did not fall within the scope of the Danish Medical Research Involving Human Subject Act (§14) (Retsinformation 2020). The study adhered to the ethical principles outlined in the Declaration of Helsinki.

## Conflicts of Interest

The authors declare no conflicts of interest.

## Data Availability

The data that support the findings of this study are available from the corresponding author upon reasonable request.
